# Benign Lymphoid Polyp of the Rectum Diagnosed and Treated With Endoscopic Submucosal Resection With a Ligation Device

**DOI:** 10.7759/cureus.55921

**Published:** 2024-03-10

**Authors:** Tomoyuki Nishimura, Tomotaka Tanaka, Syohei Ishimaru, Keiko Arataki, Fumio Shimamoto

**Affiliations:** 1 Department of Gastroenterology, Tsuchiya General Hospital, Hiroshima, JPN; 2 Faculty of Health Sciences, Hiroshima Cosmopolitan University, Hiroshima, JPN

**Keywords:** rectal tonsil, neuroendocrine tumor, endoscopic submucosal resection with a ligation device, colonoscopy, benign lymphoid polyp of the rectum

## Abstract

Benign lymphoid polyps of the rectum, also termed "Rectal tonsil" or "Pseudolymphoma," are submucosal tumor-like growths with localized hyperplasia of the lymphoid follicles and are often discovered incidentally during colonoscopy. Its diagnosis and differentiation from other submucosal tumors pose challenges owing to their similar endoscopic features. A 72-year-old woman presented with a positive fecal occult blood test, which led to the discovery of a 10-mm lower rectal tumor resembling a neuroendocrine tumor during colonoscopy. Upon closer examination, the lesion had a yellow submucosal appearance with dilated capillaries. Endoscopic submucosal resection with a ligation device (ESMR-L) was performed because the patient preferred immediate removal. Histopathological examination revealed lymphocytic infiltration with germinal center-containing lymphoid follicles, confirming the diagnosis of benign lymphoid polyp. Benign lymphoid polyps are often difficult to differentiate from carcinoid tumors and malignant lymphomas because the endoscopic findings are similar. Although preoperative endoscopic ultrasonography aids localization and characterization, definitive differentiation remains elusive and necessitates complete lesion resection. ESMR-L is a viable approach for diagnostic accuracy and therapeutic intervention, offering advantages in terms of procedural efficiency and patient care, particularly in cases involving submucosal rectal lesions.

## Introduction

A lymphoid polyp of the rectum is a benign submucosal tumor-like growth associated with localized hyperplasia of lymphoid follicles in the submucosa. Lymphoid follicular hyperplasia is thought to be caused by reactive changes due to chronic stimuli. Histologically, it consists of lymphoid follicles with germinal centers in the mucosal and submucosal layers. The mucosal epithelium is atrophic, with a loss of intrinsic glandular ducts and erosions. It is also called "rectal tonsil," "benign lymphoma," or "pseudolymphoma," and is relatively common in middle-aged and older women [1-4]. Although it is sometimes discovered through bloody stools, most cases are discovered incidentally during colonoscopy and are often asymptomatic. It is difficult to differentiate it from other submucosal tumors based on endoscopic findings. Occasionally, it is difficult to accurately diagnose even with biopsy findings. In this report, we describe a case of a benign lymphoid polyp of the rectum, diagnosed and treated with endoscopic submucosal resection with a ligation device (ESMR-L).

## Case presentation

**Figure 1:**
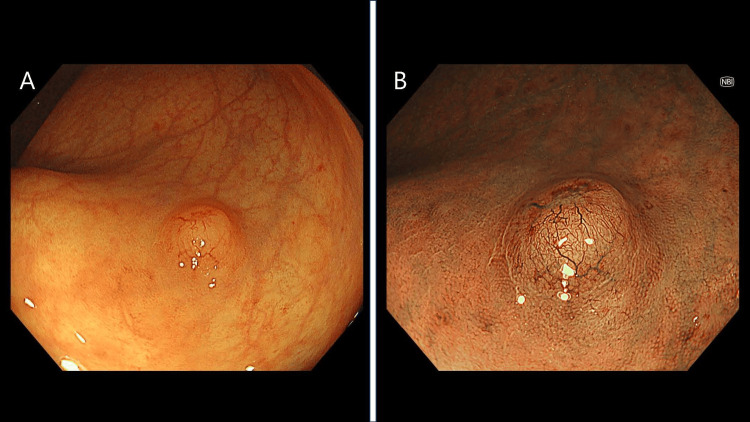
Colonoscopy A: The lesion endoscopically looked like a yellow submucosal tumor covered with normal mucosa. B: Narrow-band imaging (NBI) with magnification showed dilated capillaries on the tumor surface.

A 72-year-old female visited her family doctor after a positive fecal occult blood test during a physical examination. Colonoscopy revealed a 10-mm tumor in the lower rectum. The patient was referred to our hospital for diagnosis and treatment. Endoscopically, the lesion was a yellow submucosal tumor covered with normal mucosa. Narrow-band imaging with magnification revealed dilated capillaries on the tumor surface (Figure 1).

**Figure 2:**
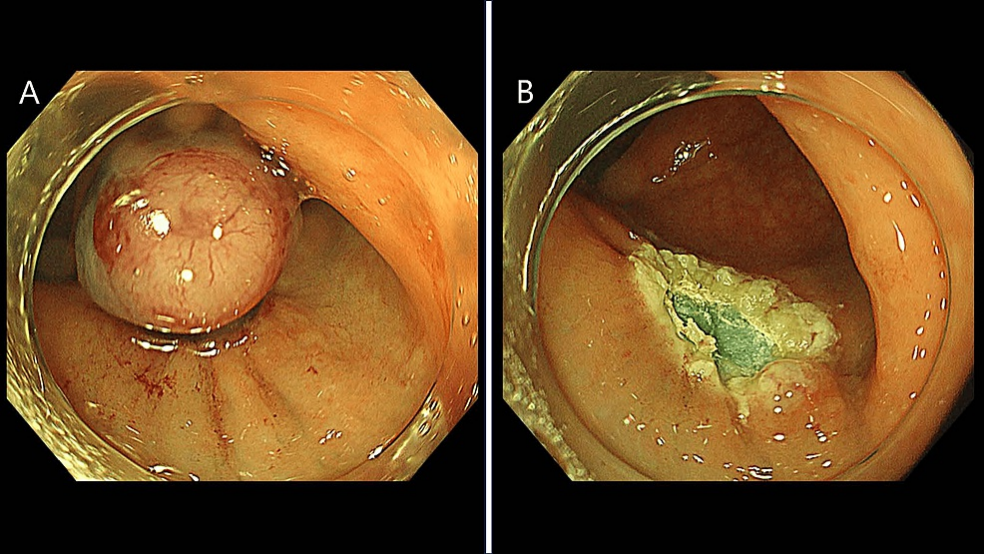
Endoscopic submucosal resection with a ligation device (ESMR-L) A: After injection into the submucosa, the lesion was aspirated using a ligature device and ligature. B: A snare was placed under the ligature band and the lesion was excised.

No other colonic lesions were observed. She had no significant medical history and had never undergone colonoscopy previously. Physical examination revealed no other abnormalities. Tumor markers, such as Carcinoembryonic Antigen and Carbohydrate Antigen 19-9, were not elevated. A tissue biopsy was suggested to the patient; however, because the patient wanted earlier resection, ESMR-L was performed for diagnostic and treatment purposes (Figure 2).

**Figure 3:**
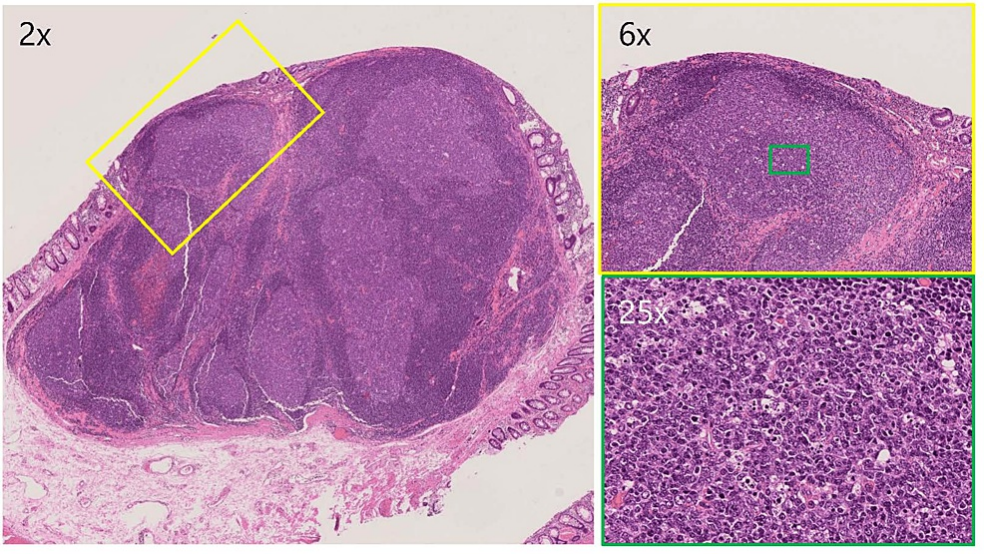
Hematoxylin and eosin (H&E) Lymph follicles had germinal centers, and tingible body macrophages were scattered at magnifications of 2×, 6×, and 25×. The lymph follicles were reactive lymphoid tissue with no cellular atypia.

The excised specimen showed lymphocytic infiltration with lymphoid follicle formation extending from the mucosa to the submucosa. The lymphoid follicles had germinal centers and scattered macrophages (Figure 3).

**Figure 4:**
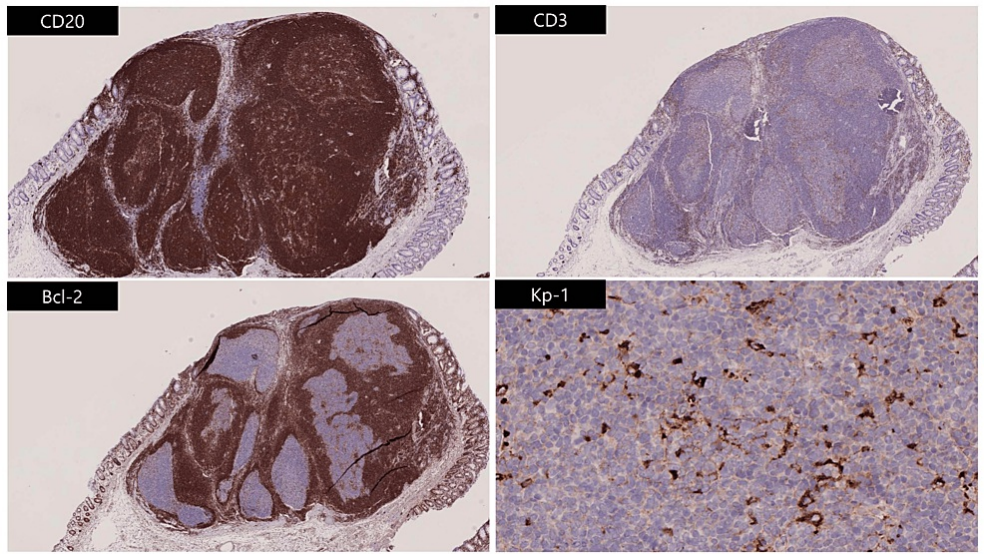
Immunohistological staining Immunohistological staining was CD20 positive, CD3 negative, and Bcl-2 negative in germinal centers; Kp-1 showed numerous macrophages.

Immunohistochemistry revealed reactive lymphoid follicles and a follicular type of proliferation with CD20-positive B-cells and CD3-positive intraepithelial T lymphocytes (Figure 4).

There was no evidence of monoclonal expansion of B-cells or cellular dysmorphism, and a benign lymphoid polyp was diagnosed.

## Discussion

Benign lymphoid polyps often occur in the lower rectum. They were reported by Helwig in 1951, and since then, they have been reported in large numbers, mainly in Europe and the United States [5]. However, there are no clear guidelines on the diagnosis and treatment of lymphoid polyps. Differential diagnoses include carcinoid tumors and malignant lymphomas; however, it is often difficult to distinguish submucosal tumors grossly, as reported by Cornes et al [1]. As previously reported, endoscopic findings of rectal neuroendocrine tumors are small lesions of 10 mm or less, often presenting as submucosal tumor-like, aphthous elevations, and the tumor has a yellowish tone and is often accompanied by dilation of surface blood vessels [6,7]. Therefore, we initially suspected a neuroendocrine tumor based on the endoscopic findings. Although preoperative endoscopic ultrasonography (EUS) was not performed in this case due to the medical facilities, it has been reported that EUS is useful for preoperative examination because the lesion is mostly localized in the third layer and has a uniform interior with low echogenicity [8,9]. EUS is highly useful for the qualitative diagnosis of tumors and evaluation of the site of origin and should be added as a preoperative test if medical facilities are available. However, malignant lymphomas can have many different morphologies, and it is difficult to reliably differentiate from benign lymphoid polyps even with EUS. In addition, not all facilities necessarily have EUS, and adding EUS testing at different facilities can increase the patient burden and healthcare costs.

These masses, with a predominantly submucosal location, are often difficult to definitively diagnose on biopsy, and complete en bloc resection of the lesion for diagnostic treatment and histopathological evaluation is preferred. However, there has been concern that endoscopic mucosal resection (EMR) may result in positive resection margins. Therefore, some facilities perform endoscopic submucosal dissection (ESD). However, this leads to increased medical costs and treatment time, resulting in an increased burden on patients. ESMR-L was developed to secure vertical and horizontal margins for endoscopic resection of submucosal tumors and to prevent perforation, thereby enhancing safety [10,11]. In the ESMR-L procedure, the lesion is aspirated, the submucosa is elevated, and the area is strangulated with band ligation and resected using a snare, thereby achieving free vertical margins. Matsuno et al. reported that both ESMR-L and ESD showed similar high complete resection rates for small rectal neuroendocrine tumors [12]. They concluded that considering the shorter procedure time and shorter hospitalization period, ESMR-L is the more efficient treatment method, especially for less-experienced endoscopists. ESMR-L is considered useful not only for the endoscopic treatment of rectal submucosal tumors but also for diagnosis because it guarantees resection margins and is a simple method.

## Conclusions

Benign lymphoid polyps, which are asymptomatic and often found incidentally, may be difficult to differentiate from other submucosal tumors. ESMR-L is a viable approach in terms of both diagnostic accuracy and therapeutic intervention, especially in cases of submucosal lesions. It has advantages in terms of procedural efficiency and patient care. This case highlights the significance of considering benign lymphoid polyps in the differential diagnosis of rectal tumors. It underscores the utility of ESMR-L as a safe and efficient management approach. Because it does not require frequent endoscopy, ESMR-L is a diagnostic treatment option from the standpoint of medical resources. Based on the above, we recommend diagnostic treatment with ESMR-L for rectal submucosal tumors such as the present case.
